# Interlocutors’ Age Impacts Teenagers’ Online Writing Style: Accommodation in Intra- and Intergenerational Online Conversations

**DOI:** 10.3389/frai.2021.738278

**Published:** 2021-08-30

**Authors:** Lisa Hilte, Walter Daelemans, Reinhild Vandekerckhove

**Affiliations:** Department of Linguistics, University of Antwerp, Antwerp, Belgium

**Keywords:** accommodation, intergenerational communication, intragenerational communication, adolescents, age, online communication, mirroring

## Abstract

The present study examines how teenagers adapt their language use to that of their conversation partner (i.e., the linguistic phenomenon of accommodation) in interactions with peers (intragenerational communication) and with older interlocutors (intergenerational communication). We analyze a large corpus of Flemish teenagers’ conversations on Facebook Messenger and WhatsApp, which appear to be highly peer-oriented. With Poisson models, we examine whether the teenage participants adjust their writing style to older interlocutors. The same trend emerges for three sets of prototypical markers of the informal online genre: teenagers insert significantly fewer of these markers when interacting with older interlocutors, thus matching their interlocutors’ style and increasing linguistic similarity. Finally, the analyses reveal subtle differences in accommodation patterns for the distinct linguistic variables with respect to the impact of the teenagers’ sociodemographic profiles and their interlocutors’ age.

## Introduction

Various sociolinguistic studies have examined the correlation between people’s age and their language use, both in on- and offline contexts (e.g., Pennebaker and Stone 2003; Tagliamonte and Denis 2008; Varnhagen et al., 2010; Herring and Kapidzic, 2015; Verheijen, 2018). For instance, youths’ language has been shown to be more innovative, creative, and non-standard (Eckert, 1997, 163; Androutsopoulos, 2005, 1,499; De Decker, 2014, 44) as well as more emotionally expressive (Argamon et al., 2009; Schwartz et al., 2013, 9). In addition, linguistic differences emerge between youths/teenagers of different ages too (Hilte et al., 2018; Hilte et al., 2020c) (see below for an overview).

Apart from speakers’ or authors’ own age, their interlocutor’s age also has a linguistic impact. The adaptation of one’s language use to that of others is called accommodation. Within the field of accommodation, ample research has been conducted on intra-versus intergenerational communication, i.e., communication among peers versus among people of different ages (Williams and Nussbaum, 2001; McCann et al., 2005; Giles and Gasiorek, 2011). In intergenerational conversations, patterns of under- and overaccommodation have been attested repeatedly (Williams and Nussbaum, 2001, 85, 89; Giles and Gasiorek, 2011, 233–234) (see below for an overview).

However, studies on intergenerational communication often focus on the elderly rather than on younger adults (e.g., Ytsma and Giles, 1997; Williams et al., 2005), and they rarely include teenagers. Consequently, very little is known about how teenagers communicate with older interlocutors. Furthermore, spontaneous informal online conversations are under-represented in accommodation research. This is quite surprising in view of the fact that in intergenerational contexts, the online setting is particularly interesting, since many youths are “digital natives” (having grown up with digital media, Frey and Glaznieks, 2018), whereas certain older people are not. Therefore, when analyzing accommodation patterns in online intergenerational interactions, it is worth focusing not only on “traditional” language features (e.g., markers of oral vernacular), but also on features typical of digital media (e.g., emoji). The present paper aims to fill these gaps. We will investigate whether youths adapt their writing style in online interactions depending on their interlocutor’s age. This research complements our previous work on gender- and education-related accommodation in teenagers’ online conversations (Hilte et al., 2020b; Hilte et al., forthcoming).

The paper is structured as follows: First, we present an overview of related research and lay out the research questions. Next, we introduce the materials and methods. Finally, the results are reported and discussed.

## Related Research

Below, we present an overview of previous findings on adolescents’ online writing style and on accommodation in intra-versus intergenerational settings. In the latter section, we also address the main research questions that this paper aims to answer.

### Adolescents’ Online Writing Style

Age is a naturally dynamic socio-demographic variable, and as people grow older, their language use evolves with them, often aligning with physical, psychological, or social-developmental changes (Pennebaker, 2011, 60–61; Williams and Nussbaum, 2001, 5). This linguistic evolution affects both content, with certain topics gaining and others losing importance as people grow older (Argamon et al., 2007; Schwartz et al., 2013) and style, with e.g., shifting preference patterns for certain types of function words (Argamon et al., 2007, 2009; Pennebaker, 2011) and with language use of older people reflecting increased cognitive complexity (Pennebaker, 2011, 62; Pennebaker and Stone, 2003, 295–296).

We will now zoom in on teenagers’ language use, and their online discourse in particular, which is the focus of the present paper. It is widely accepted that creativity, linguistic innovation and non-standard language use peak during puberty (Eckert, 1997, 163; Androutsopoulos, 2005, 1,499; De Decker, 2014, 44). This has been linked to both (linguistic) rebellion and to teenagers being “relatively free of responsibilities and normative pressures from the linguistic market” (Wagner, 2012, 375). However, when reaching adulthood, people tend to turn away from (youth) slang in favor of more conservative or mainstream language patterns, which has been linked to the responsibilities that come with adult life and a decreased preoccupation with self-definition (Wagner, 2012, 375, 379). This pattern of age grading can be found in online discourse too, with younger chatters and especially teenagers using (and self-reporting to use) more prototypical markers of online writing than older chatters (Argamon et al., 2009; Schwartz et al., 2013, 9; Oleszkiewicz et al., 2017; Prada et al., 2018, 1929). Many innovations in adolescent talk are said to “primarily serve expressive and interactive purposes” (Androutsopoulos 2005, 1,499), and this seems to hold for social media writing too: an important share of the stylistic features that teenagers use more frequently and evaluate more positively than adults are “expressive markers” that add emotion to a text (e.g., emoji–see also *Linguistic Variables*) (Argamon et al., 2009; Schwartz et al., 2013, 9; Verheijen, 2015, 135; Oleszkiewicz et al., 2017; Prada et al., 2018; Verheijen, 2018, 127). Note that most prototypical markers of (especially teenagers’) instant messaging can be linked to one of three “maxims” (implicit rules of linguistic conduct) of informal online writing: the principles of expressive compensation, orality, and brevity (Androutsopoulos, 2011, 149; Thurlow and Poff, 2013, 176). These principles concern writing as you speak, typing/interacting fast, and typographically compensating for non-verbal emotional cues from face-to-face interactions. Linguistic features belonging to these three maxims will be the focus of the present paper (see *Linguistic Variables* for an extensive description and illustrations). Still, an important nuance concerning (online) teenage talk is that adolescence is no homogeneous linguistic period with respect to style (content-wise, younger and older teenagers’ online discourse does reveal the same prominent topics; Hilte et al., 2020a). The so-called “adolescent peak” phenomenon has been attested repeatedly, with non-standard language use and linguistic nonconformity increasing in early adolescence, peaking mid-puberty (around the age of 15–16) and then decreasing as adulthood comes closer (De Decker and Vandekerckhove, 2017, 277; Holmes 1992, 184). In informal social media writing, younger teenagers appear to insert more non-standard features than older teenagers: this holds for both “traditional” vernacular (e.g., regional language features) and “new” (digital media-specific) vernacular (e.g., emoji) (Hilte et al., 2018; Hilte et al., 2020c; Verheijen, 2015, 135–136; 2016, 283, 285; 2018, 127). These findings suggest changing attitudes with age (i.e., a decrease in appreciation) concerning deviations from the formal linguistic standard: while younger adolescents seem to consider them as cool and may use them for the expression of peer group belonging and for personal identity construction (De Decker and Vandekerckhove, 2017, 278; Verheijen, 2015, 129), older adolescents might see them as “somewhat childish” (Verheijen, 2015, 135). Strikingly, this age pattern with respect to non-standard language use was found to be stronger for teenage girls than for boys, suggesting that “girls and boys derive different prestige from standard and non-standard markers in their late teens, and that especially girls turn away from non-standard markers (to some extent)” (Hilte et al., 2020c, 194). We note that this pattern of interaction between age and gender in online writing echoes older sociolinguistic findings on offline language (Trudgill, 1983; Eisikovits, 2006).

Furthermore, teenagers’ age impacts more general text features in informal online writing too (e.g., average sentence length–see Hilte et al., 2020a), but this falls outside the scope of the present study.

Finally, teenagers prove to be well aware of the sociolinguistic patterns described above. In one of our previous studies, they performed very well in an age-detection task, and their intuition on younger versus older adolescents’ writing styles was quite accurate (Hilte et al., 2019). Consequently, age accommodation (see below) might not only be the result of subconscious pattern matching but could also consist of more conscious adaptations based on actual awareness of sociolinguistic patterns.

### Accommodation in Intra- and Intergenerational Communication

Linguistic accommodation^1^ is the adaptation of one’s communicative behavior to (that of) one’s conversation partner. The sociolinguistic framework “Communication Accommodation Theory” (CAT) serves as our main point of reference. CAT considers the main goals of accommodation to be facilitating interaction and regulating social distance among interlocutors (Dragojevic et al., 2015, 10). Common adaptation strategies are convergence and divergence, resulting in communicative behavior that is more respectively less similar to others’ (Giles and Ogay 2007, 295–296). While divergence is generally evaluated more negatively and convergence more positively, full convergence is rarely desired, since there seem to exist individually and socio-culturally determined optimal levels of similarity (Dragojevic et al., 2015, 13, 15; Burgoon et al., 2017). Overaccommodation can even be perceived as parody (Jones et al., 2014, 457) or as patronizing (see below).

While the inclination to adapt one’s language use to that of others is in part individually determined (Jones et al., 2014; Xu and Reitter 2015), some robust accommodative patterns have been attested relating to interlocutors’ socio-demographic or psychological profiles. We will zoom in on our social variable of interest: age. Intergenerational communication (i.e., between interlocutors of different ages/generations) can present interlocutors with an interactive challenge: since people of different ages may live in very different (physical, cognitive, or social) contexts (see also Krohn 2004), they are sometimes considered as belonging to different “developmental cultures”, which links intergenerational and intercultural communication (Williams and Nussbaum 2001, 7).

Intergenerational communication is often described as problematic, uncomfortable, or dissatisfying for the different parties (Giles and Gasiorek 2011, 233). These negative perceptions are related to the accommodation patterns observed in such conversations: (mostly) underaccommodation by older interlocutors versus reluctant accommodation or different types (e.g., verbal versus nonverbal) of overaccommodation by younger interlocutors (Williams and Nussbaum 2001, 85, 89; Giles and Gasiorek 2011, 233–234). Underaccommodation is an issue if people fail to adjust their communicative behavior to that of others (Giles and Gasiorek 2011, 240). Examples of older people’s underaccommodation relate to speed and expressive behavior: e.g., older people may react slower and make little eye contact when interacting with younger people (Williams and Nussbaum 2001, 89–90). The opposite phenomenon of overaccommodation consists in overshooting the communicative behavior that is required for successful and smooth interaction (Giles and Gasiorek 2011, 234, 240). Overaccommodation directed at the elderly often consists in “adjusting […] communication to compensate for perceived physical or psychological deficits of an older adult” (Giles and Gasiorek 2011, 234), e.g., oversimplified, slow, or excessively loud talk (Williams and Nussbaum 2001, 108, 111). This type of overaccommodation–which is referred to as patronizing talk (Giles and Gasiorek 2011, 234)—may be more accepted in institutional settings such as hospitals, but is generally associated with negative perceptions, disempowerment and lowered self-esteem for the elderly, and even self-stereotyping (i.e., elderly adopting stereotypes about old age that are made salient to them in interactions with younger people) (Williams and Nussbaum 2001, 107, 109; Giles and Gasiorek 2011, 235–236). Intergenerational interactions (and especially overaccommodation patterns) are often impacted by people’s assumptions and/or stereotypes about age and age-bound communicative styles (Williams and Nussbaum, 2001, 3, 110; Giles and Gasiorek, 2011, 238). Over time, such stereotypes may gain strength by “trigger[ing] a negative feedback cycle that results in overaccommodative talk and, ultimately, in a reinforcement of age stereotypes” (Giles and Gasiorek, 2011, 238). The (linguistic) treatment of people in terms of stereotypes is considered to be stronger when interlocutors focus more on their respective group memberships (e.g., in terms of age) than on individuals’ qualities, e.g., when interlocutors have little (other) personal information about each other (Williams and Nussbaum, 2001, 9–10).

It is important to note that large age differences between interlocutors often imply differences in social position, too. These differences are impactful, since CAT predicts shifts towards interlocutors with greater “power” (Dragojevic et al., 2015, 4), as people in the lower power position are presumed (and have been attested) to desire the other’s approval more than vice versa (Muir et al., 2016, 477). Consequently, age and social power might interact with respect to accommodation patterns in intergenerational communication (see *Materials*). Such interactions or “confusion” between social variables emerges in related research too. For instance, de Siqueira and Herring (2009) examine (online) conversations among doctoral students and their supervisor and suggest that age, gender, and social hierarchy might affect accommodation simultaneously.

This contribution aims to fill certain gaps in accommodation research. Studies on intergenerational communication rarely include teenagers–the target group of the present paper–even though the desire to obtain social approval (a driving force behind accommodation according to CAT) might be stronger among teenagers than adults, as this group is often driven by a “need of acceptance” and “fear of rejection” (Taylor, 2001, 298). In addition, older interlocutors included in intergenerational studies are often truly “elderly”. Consequently, communication between teenagers and e.g., people in their thirties are under-researched. Finally, accommodation is mostly analyzed in spoken face-to-face dialogue. While it has been studied to which extent these findings translate to online communication (Scissors et al., 2008; 2009; Riordan et al., 2013; Doyle et al., 2016), as of yet, there are no large-scale studies on accommodation in online corpora that truly mirror spontaneous face-to-face interactions, as studies are either carried out on small corpora (e.g., Wolf, 2000; de Siqueira and Herring, 2009), on public, asynchronous conversations (e.g., Dino et al., 2009; Danescu-Niculescu-Mizil et al., 2011; Doyle et al., 2016), or on conversations between strangers and/or in lab-based settings (e.g., Niederhoffer and Pennebaker 2002; Scissors et al., 2008; 2009; Gonzales et al., 2010; Muir et al., 2017). We note that our previous work on gender- and education-based accommodation, which this contribution complements, is an exception (Hilte et al., 2020b; Hilte et al., forthcoming). The present paper focuses on accommodation in teenagers’ instant messages: do teenagers significantly adapt their online writing style to that of their (older) conversation partners? Or in other words: do teenagers adopt different styles depending on their interlocutors’ age?

Finally, in intergenerational communication, the online medium is particularly interesting, since younger generations are often “digital natives” (having grown up with digital media, Frey and Glaznieks, 2018) and highly “computer literate” (Krohn, 2004, 326), whereas certain older people are not. Age has indeed been shown to negatively correlate with (self-reported) use of digital media (Prada et al., 2018, 1927). This is an example of how people of different ages may also show “differences that reflect collective changes in cultures” (Pennebaker and Stone, 2003, 293–294). In the present contribution, we will therefore inspect accommodation with respect to features typical of social media writing, in order to verify whether teenagers construct their digital discourse with the age of their interlocutor in mind.

## Materials and Methods

Below, we discuss the dataset (*Materials*) and the methodology of the analyses (*Methods*).

### Materials

The corpus consists of 347,504 private instant messages (2 million tokens^2^) produced by 1,203 Flemish teenagers in Dutch on Facebook Messenger and WhatsApp, mainly between 2015–2016. At the time of collection, the teenagers were secondary school students aged 13–20. They nearly all lived in the province of Antwerp, in the center of Flanders (i.e., Dutch-speaking northern Belgium). All participants attended one of the three main types of Belgian secondary education, ranging from the theory-oriented general education, where students are prepared for higher education, to the practice-oriented vocational education, where students are prepared for specific, often manual, professions. Technical education holds an intermediate position in terms of theory and practice (FMET, 2018, 10).

We visited schools and invited pupils to voluntarily donate (parts of) their chat conversations (produced out of the school context and before our visit). For WhatsApp, the students could easily export entire conversations *via* the app: these conversations were automatically converted to plain text files and attached to an e-mail. While this plain text format kept all text, including special characters such as emoji, the students could opt to automatically delete all media files (e.g., gifs, pictures, video or audio files inserted in the chat conversations). All remaining media files (only applicable if the students did not select the delete-option) were removed by us, since their analysis falls outside the scope of our research. For Facebook Messenger, the students were instructed to copy their conversations from the Facebook website and paste them to a submission website that we created. These pasted texts were converted to a plain text format too, from which all media files were removed, but in which all text and special characters were kept. The participants also provided the following metadata (*via* email or *via* a form on the submission website): their age, gender, and educational track. The messages uttered by the participants’ conversation partners were deleted (unless the latter were teenagers that participated in the process of data collection themselves), but the teenage participants did provide age information on their interlocutors (so we know which messages were uttered by teenagers interacting with peers, versus with people from other age groups). The pupils’ (and for minors, also their parents’) consent was asked to store and linguistically analyze their texts after anonymization. We note that this corpus is a subset of a larger dataset (see Hilte, 2019), selected on its relevance for the present study.^3^

Our socio-demographic variable of interest is age. All participants (who donated chat conversations and whose utterances we can linguistically analyze) are teenagers. In the analyses, we will distinguish participants in early adolescence (aged 13–16) and in later adolescence (aged 17–20), as teenagers’ non-standard language use does not evolve linearly, but “peaks” mid-puberty (see above). We note that a same teenage participant can occur in the corpus in both age categories (as a younger as well as an older adolescent), in case they donated both older parts of their chat history (i.e., when they were still a young teenager) and more recent parts (i.e., as an older teenager). In addition to the teenage participants’ own age, we also have age information about their interlocutors, i.e., the people that the teenagers correspond with online. Recall that these people’s chat utterances were deleted from the corpus, but that their age was provided by the participating teenagers. Consequently, when analyzing the teenage participants’ language use, we can distinguish between conversations in which teenagers talk to other teenagers (13–20) versus to twenty-somethings (20–30) versus to people over thirty (30+). Finally, each conversation was labeled based on the generation “gap” between its interlocutors: in “intragenerational” talks the teenage participants interact with other teenagers (13–20), and in “intergenerational” talks they interact with older people (20+). Table 1 presents an overview of these distributions in the corpus.

**TABLE 1:** Distributions in the corpus with respect to interlocutors’ age^4^.

Social variable	Variable level	Conversations	Teenage participants	Tokens (uttered by teenagers)
Author age	Young teenager (13–16)	906	773	1,017,408 (51%)
	Older teenager (17–20)	848	605	973,377 (49%)
Interlocutor age combinations	Teenagers only (13–20)	1,384 (93%)	1,200	1,951,889 (98%)
	Teenagers (13–20) and twenty-somethings (20–30)	40 (3%)	23	23,439 (1%)
	Teenagers (13–20) and people over thirty (30+)	61 (4%)	26	15,457 (1%)
Generation gap	Intragenerational (only teenagers)	1,384 (93%)	1,200	1,951,889 (98%)
	Intergenerational (teenagers + older generations)	101 (7%)	47	38,896 (2%)
Total		1,485	1,203	1,990,785

As Table 1 shows, the vast majority (93%) of the conversations are intragenerational (including only teenagers), and only a small portion (7%) are intergenerational (including both teenagers and older people)—both conversational contexts will be examined in this paper. So the teenagers predominantly chat with their peers. This fits inside the fairly recent phenomenon of a “peer society”: “[a]s the boundaries between the various stages of adulthood, adolescence, and childhood were strengthened perceptually in popular social consciousness and in social practice, it became almost natural to segregate children and adults into same-age peer groupings” (Williams and Nussbaum 2001, 33). Williams and Nussbaum also reflect on the changes in demographic and family structures that led to this evolution and conclude that a peer-centered society implies “increasingly minimal contact […] between younger and older generations” (2001, 36). While at first glance, the distributions in Table 1 seem to show this minimal contact across generations persisting in an online setting, it is crucial to keep in mind that our corpus contains instant messages only. Other (e.g., real-life) forms of intergenerational contact are simply not included. What we can safely conclude from this corpus is that adolescents use instant messaging media predominantly as “peer-platforms”. While this dominance of peer-to-peer interactions in the corpus is an interesting finding, it obviously raises challenges too, as it results in a skewed dataset. Since the less frequent intergenerational encounters still contain a reasonable amount of data, we can proceed with the statistical analyses (see below). However, in future work, a sample with a larger share of intergenerational conversations may allow for a more robust examination of our research questions.

As mentioned above, the relationship between conversation partners might have an impact on accommodative behavior too. This variable was manually annotated per conversation in the corpus: a human annotator read the (anonymized) interactions and, whenever possible, determined the relationship between the conversation partners based on the content of the interaction. While the relationships between interlocutors vary from very to not at all intimate, none of the participants chat with strangers. The vast majority of intragenerational conversations (i.e., all-teenagers) include teenage interlocutors who are friends (78%). Others are lovers (4%) or relatives (2%). The relationship for the remaining talks could not be determined by the annotator (as it was either unclear or ambiguous, or the conversation was simply too short to obtain an accurate image of the relationship between the conversation partners). A similar distribution emerges for intergenerational talks with the smallest age gap, i.e., interactions between teenagers and twenty-somethings: these interlocutors are mostly friends too (75%), followed by lovers (10%) and relatives (10%). However, in conversations between teenagers and people over thirty, we can see a shift in the type of relationships. Most interlocutors are relatives (59%), followed by friends (30%). Moreover, in this category hierarchical “power” relationships emerge (11%), such as a relationship between sports coach and pupil or an employment relationship. Romantic relationships are absent in this part of the dataset. These sociological (“power”) differences may be a source of asymmetric accommodation, with the interlocutor in the “lower” position converging more strongly to the other than vice versa (see above). However, data sparsity and cases of so-called “complete separation”—i.e., certain relationships being too infrequent or completely absent, respectively, in either the intra- or intergenerational settings–prevent the systematic inclusion of power as a confounding factor in the statistical analyses. This issue will be particularly hard to solve in future work, even with an updated or entirely new dataset, since age and power are often intertwined: it may be challenging to collect naturalistic data from e.g., pairs of teenagers with power differences, and/or pairs of a teenager and a (much) older interlocutor without any power imbalances.

With respect to other potentially confounding factors, we note that the combination of interlocutors’ gender (i.e., same-versus mixed-gender interactions) is known to impact youths’ online writing style too (Hilte et al., 2020b). However, we expect no interference from this factor since nearly identical distributions (i.e., a much larger share of same-gender talks compared to mixed-gender conversations) emerge in both the intra- and intergenerational parts of the corpus. Apart from gender, the combination of interlocutors’ educational track (i.e., same-versus mixed-education talks) has been found to influence accommodative behavior too (Hilte et al., forthcoming). Here we see a parallel with the shifting relationships as older people are involved (see previous paragraph): a strong discrepancy in age often implies a shift in education too. All talks between teenagers and older people (20+) are labeled mixed-education, because students from secondary education chat with people in higher education or people who have already acquired a degree for higher education, whereas the majority of interactions among teenagers are same-education, meaning that they are in exactly the same track in secondary education. Finally, imbalances regarding the authors’ (and not the interlocutors’) gender and educational track and concerning the number of interlocutors in a conversation may act as confounding factors. Therefore, these variables are included in the research design (see *Method*). The distribution of these categories in the dataset is shown in Table 2. We note that the number of interlocutors was operationalized as a binary variable: we distinguish one-on-one chats (two interlocutors) from group chats (more than two interlocutors).

**TABLE 2:** Distributions in the dataset with respect to confounding factors.

Variable	Variable levels	Participants	Tokens
gender	girls	637 (53%)	1,368,641 (69%)
	boys	566 (47%)	622,144 (31%)
educational track (in secondary school)	General (theory-oriented)	534 (44%)	615,548 (31%)
	Technical (hybrid)	355 (30%)	950,027 (48%)
	Vocational (practice-oriented)	314 (26%)	425,210 (21%)
Total	—	1,203	1,990,785
**Variable**	**Variable levels**	**Conversations**	**Tokens**
number of interlocutors	one-on-one (2 interlocutors)	1,283 (86%)	1,451,864 (73%)
	group chat (>2 interlocutors)	202 (14%)	538,921 (27%)
Total		1,485	1,990,785

### Methods

Below, we present the linguistic variables and the methodology for the analyses.

#### Linguistic Variables

As mentioned above, many prototypical markers of instant messaging can be linked to one of three “maxims” (implicit rules of linguistic conduct) of informal online writing: the principles of expressive compensation, orality, and economy (Androutsopoulos, 2011, 149; Thurlow and Poff, 2013, 176). Below, we describe these maxims along with their related features, illustrated with examples from the dataset. The selection of the features was based on related research (e.g., Varnhagen et al., 2010; Verheijen, 2018) and on our own previous work, in order to facilitate systematic comparison between our current and previous findings (Hilte et al., 2020b; Hilte et al., 2020c; Hilte et al., forthcoming).

The principle of expressive compensation accounts for a wide range of (mostly typographic) strategies to compensate for the absence of certain expressive cues in written communication, such as facial expressions, volume, or intonation. We include the following features^5^:

- emoticons/emoji:
  e.g., 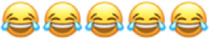, 
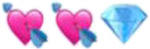

- expressive repetition of letters and punctuation marks:
  e.g., *Ik ben zoooo blij!!!* (“I am soooo happy!!!”)
- words/phrases rendered in capital letters (“allcaps”):
  e.g., YES
- typographic rendering of kisses and/or hugs:
  e.g., Dankje xxxx (“Thank you xxxx”)
  e.g., tot straks! xoxo (“See you later! xoxo”)
- onomatopoeic rendering of laughter:
  e.g., haha, whahahhaha
- combinations of question and exclamation marks:
  e.g., Nee echt?! (“No, seriously?!”)

The orality principle concerns speech-like writing: the register in informal written online interactions is often to a large extent “conceptually oral”, reflecting typical speech patterns rather than classical written communication. In our corpus of Flemish teenagers’ Dutch instant messages, this principle results in the insertion of different kinds of non-standard Dutch lexemes and non-standard grammar which render the written utterance more speech-like:

- dialect/regiolect words:
  e.g., wij hadden ambras (std. Dutch: wij hadden ruzie, “we had a quarrel”)
- informal/colloquial words or slang:
  e.g., echt brak (std. Dutch: echt slecht, “really bad”)
- orthographic renderings of non-standard pronunciation or morphosyntax:
  e.g., Dee wilt ni da gij gaat (std. Dutch: Hij wil niet dat jij gaat, “He does not want you to go”)
  Furthermore, Flemish teenagers often insert English words or phrases that are part of Dutch adolescent speech in their online texts. We include as features:
- English words rendered in their “original” form:
  e.g., Knappe dude (“Handsome dude”)
- English words adapted to Dutch (in terms of e.g., spelling or morphology):
  e.g., heel naajs (“very nice”)
  e.g., suckt echt (“really sucks”)

Note that the base language in the dataset is always Dutch (entire conversations in another language were excluded). Furthermore, English loan words that have been integrated in Dutch for such a long time that they are considered part of standard Dutch vocabulary and included in Dutch dictionaries (e.g., computer), are counted as Dutch and not English.

The third and final chatspeak maxim is the principle of brevity, which covers all kinds of strategies to compress words or utterances. We include as features:

- typical chatspeak abbreviations and acronyms (none of them are standard Dutch abbreviations)
  e.g., btw (full version: “by the way”)
  e.g., wrs (full version: waarschijnlijk, “probably”)

The feature occurrences were detected and counted automatically in the dataset with *Python* scripts. The scripts’ output was compared to a human annotator’s decisions for a test set of 200 randomly selected posts (1,257 tokens). The software reached satisfying scores: an average precision of 92% (i.e., the share of detected feature occurrences that are valid) and an average recall of 88% (i.e., the share of all feature occurrences in the test set that were detected as such by the software). The scores for the individual features were also sufficiently high. Consequently, the scripts’ output is reliable and suitable for further linguistic analysis. For an extensive discussion of the feature extraction procedure, see Hilte et al. (2020c).

We previously observed distinct online writing styles for teenagers in early versus later adolescence, with younger teenagers inserting more oral, expressive and brevity features (Hilte et al., 2018; Hilte et al., 2020c). In other words, all of the prototypical markers of the genre that are related to the three maxims discussed in the present section have higher frequencies in young teens’ social media writing than in that of their older peers. Therefore, it is worth investigating whether these two age groups will show different accommodative behavior too.

Our previous research allows us to broaden the perspective: we will compare age-related accommodation to the accommodation patterns related to gender and education that we attested in previous studies. Strikingly, we observed significant mirroring or convergence with respect to interlocutors’ gender and education for expressive markers only. In other words, the distinction between the three types of features proved essential for detecting and explaining accommodation in the discourse of these teenagers. Consequently, for the sake of comparability, the present study also investigates–apart from the main research question, i.e. whether teenagers adapt their online writing style to that of older interlocutors (see above)—which feature set is more susceptible to accommodative change with respect to interlocutors’ age. Will expressive features prove to be most salient once again or not?

Finally, note that in related work, these three sets of digital features–expressive, oral, and brevity markers–are sometimes grouped together and labeled “textisms” (e.g., Adams et al., 2018; Verheijen, 2015; 2018). In the present paper, we treat them as three separate sets of variables, since we previously observed consistently different sociolinguistic patterns per feature set with respect to the impact of both author and interlocutor profiles (Hilte et al., 2020b; Hilte et al., 2020c; Hilte et al., forthcoming). Although many textisms seem to replace face-to-face nonverbal cues to some extent, there is an important distinction: textisms, as opposed to their face-to-face counterparts, are often^6^ inserted deliberately (Adams et al., 2018, 475). Communication Accommodation Theory covers both conscious/deliberate and unconscious/automatic adjustment (Dragojevic et al., 2015). If the teenagers in the corpus modify their use of textisms when interacting with older interlocutors, the present paper will offer an example of more deliberate accommodation, i.e., adjustment of more intentional language features (see also Adams et al., 2018).

#### Method

We will statistically model the participants’ language use in intra- and intergenerational conversations, in search for accommodation patterns. Our approach to accommodation is of a quantitative nature: we analyze which features’ frequency significantly increases or decreases depending on the interlocutors’ age. Furthermore, we study accommodation from a synchronic perspective, comparing youths’ writing in different conversational settings (depending on interlocutors’ age) rather than analyzing the course of particular interactions. Diachronic analyses (including a temporal dimension) are left for future work. The present study’s methodology is similar to our previous work on gender and education accommodation (Hilte et al., 2020b; Hilte et al., forthcoming) and on social variation in youths’ online writing (Hilte et al., 2020c), which facilitates systematic comparison of our previous and current findings. Below, we describe the data preprocessing and model fitting.

For preprocessing, we created a summary of the dataset with each line or observation representing one participant in one conversation. Participants can thus occur on multiple lines (i.e., in different conversations) and conversations can be represented on multiple lines too (with each interlocutor occupying a line). We correct for these repeated observations with random effects (see below). Each line in the dataset contains the participant’s profile information (a unique, anonymous identifier as well as their age, gender, and educational track), conversational meta-information (a unique conversation identifier and the number of interlocutors), and the feature counts (i.e., the number of oral, expressive, and brevity markers for this participant in this conversation).

Next, we modeled the teenagers’ use of the three feature sets with generalized linear mixed models (GLMMs) with a Poisson distribution.^7^ These models are recommended for counts (Ismail and Jemain, 2007, 105; Harrison, 2014, 2) since the underlying Poisson distribution is considered the “simplest distribution for modeling count data” (Zeileis et al., 2008, 5). GLMMs are able to analyze the effect of different predictors simultaneously, as well as of their potential interaction. We will inspect the impact of authors’ and their interlocutors’ age on the response (i.e., counts for expressive, oral, and abbreviated markers). In addition, we include three confounding factors: the authors’ gender and educational track, and the number of interlocutors in the conversation.

As mentioned above, the models can take into account the impact of individual participants and conversations and thus correct for repeated observations, as a random effect for subject and conversation was included. This way, the models can cluster observations from one participant in different conversations, thus incorporating individual writing styles, as certain people may *always* write in a more expressive/oral/abbreviated way than others. Similarly, the models can cluster observations from different interactants in the same conversation, thus dealing with conversation-specific conventions and styles, as certain people may *always* use many expressive/oral/abbreviated markers among each other. Consequently, this random effect for conversation can incorporate *stylistic cohesion*: “(messages) belonging to the same conversation are closer stylistically than (messages) that do not” (Danescu-Niculescu-Mizil et al., 2011, 748). In order to avoid overdispersion (i.e., the variance of the response exceeding the mean–see Hilte et al., 2020c), which may result in unreliable outcomes (Ismail and Jemain, 2007, 103; Harrison, 2014, 1, 2, 17–18), we add a random effect for observation (see Harrison, 2014, 1 for a discussion of this technique). Finally, the models can handle differences in sample size between observations by adding an offset for (the logarithm of) the number of tokens per observation.

In the result section, we discuss the models that resulted in the best fit for the data. This best fit was experimentally determined through stepwise deletion of insignificant predictors.

## Results

Previous research revealed that teenagers’ age, gender, and educational track significantly influence their online writing style (Hilte et al., 2020c), and that teenagers adapt certain aspects of their writing depending on their interlocutor’s gender and educational background (Hilte et al., 2020b; Hilte et al., forthcoming). The present study aims to complement these findings by examining accommodation in intra-versus intergenerational communication. Below, we present our findings for expressiveness, orality, and brevity.

Recall that we can only examine the adjustive effort made by the teenagers and not by their older interlocutors, since the present corpus only contains data produced by teenagers. For adult (twenty plus) interlocutors, we have only information on their age category (provided by the teenagers) and on their relation to the teenager (manually annotated), but not on their writing (recall that all participants, who agreed with the terms of the study, donated text material, and provided the relevant metadata, are teenagers–see *Materials*).

### Expressiveness

**FIGURE 1:**
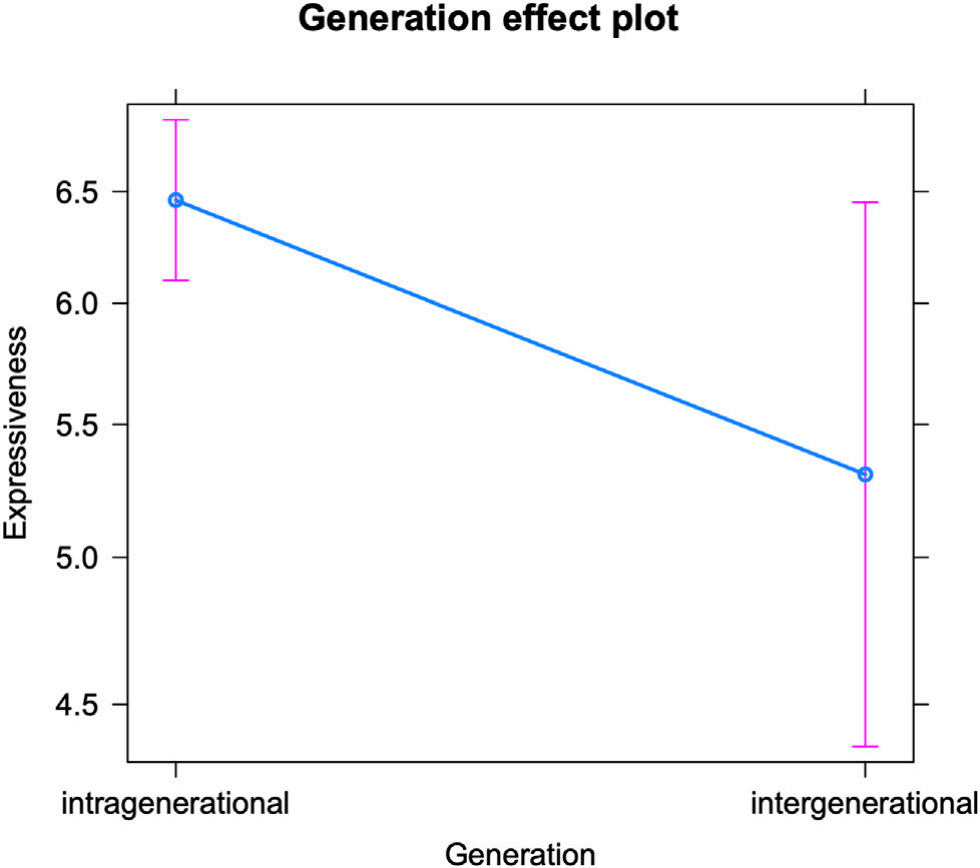
Expressive markers in intra-vs. intergenerational talks (predicted counts per 100 tokens).

Let us start by discussing teenagers’ expressive writing in intra-versus intergenerational conversations (see Table 3 for the fixed effects and Supplementary Table S1 in the appendix for the Anova). The model reveals that the teenagers in the corpus use on average 6.58 expressive markers per 100 tokens. But, as can be deduced from Figure 1, they insert significantly more expressive markers when talking to their peers (intragenerational) than to older interlocutors (intergenerational). A first potential explanation is that some intergenerational talks are simply more formal (and the relationship between interlocutors less intimate, see above). Since expressive markers do not only express emotion, but also (and far more) social proximity, it makes sense that teenagers insert them less frequently in such contexts. However, we also know that these markers are used most abundantly in (especially early) adolescence, and less frequently as youths grow older and reach adulthood (Hilte et al., 2018; Hilte et al., 2020c; Argamon et al., 2009; Schwartz et al., 2013, 9; Verheijen, 2015, 135–136; Verheijen, 2016, 283, 285; Prada et al., 2018; Verheijen, 2018, 127). Consequently, the observed decrease can also be the product of mirroring older interlocutors’ online writing style. We previously observed convergence regarding the use of expressive markers in intergender conversations (between boys and girls) (Hilte et al., 2020b), and in intereducational conversations (between students from different educational tracks) (Hilte et al., forthcoming). Consequently, the present analysis on interlocutor age confirms that expressive markers are very sensitive to accommodation. We note that Figure 1 reveals a (much) larger confidence interval for teenagers’ use of expressive features in intergenerational compared to intragenerational settings. Potential explanations concern the smaller sample size of the intergenerational subset, and the greater age variation within the intergenerational label (all interlocutors aged 20+, versus interlocutors aged 13–20 for the intragenerational label). But the larger confidence interval could also suggest that there is not one singular way in which teenagers adapt their online communication to that of older interlocutors–we will come back to this in the *Discussion*.

**TABLE 3:** Expressiveness: Fixed effects^8^.

	Estimate	Standard error	z value	*p* value
(Intercept)	−2.38049	0.05054	−47.101	<2e-16
Generation gap (intergenerational)	−0.19667	0.09913	−1.984	0.047255
Author age (17–20)	−0.32381	0.04826	−6.710	1.95e-11
Author gender (male)	−0.45397	0.05779	−7.855	4.00e-15
Author education (technical)	−0.05892	0.05425	−1.086	0.277466
Author education (vocational)	0.17319	0.06231	2.780	0.005441
Number of interlocutors (group chat)	−0.27118	0.06051	−4.482	7.40e-06
Author age (17–20): author gender (male)	0.25256	0.06960	3.629	0.000285

Furthermore, the teenagers’ linguistic adaptation is not significantly influenced by their own socio-demographic profiles, nor by the number of interlocutors in a conversation. The latter finding is in line with previously attested patterns of education-based accommodation, but differs from gender convergence, that appeared stronger in one-on-one settings (Hilte et al., forthcoming and Hilte et al., 2020b, respectively). A potential explanation is that gender-based linguistic adaptation might be of a more personal, intimate nature than age or education accommodation (for the relation/distinction between gender accommodation and flirting strategies, see Hilte et al., 2020b).

Finally, the model reveals patterns relating to the confounding factors. Since they do not concern accommodation, we will summarize them briefly. Significantly more expressive markers occur in one-on-one conversations than in group chats, which points to different conversational dynamics for these two types of interactions (see also Hilte et al., forthcoming). Next, students in the most practice-oriented educational track use most expressive markers. A final pattern concerns the interaction between the authors’ age and gender: while all teenagers use fewer expressive markers in their online discourse at an older age, this decrease is much stronger (and only significant) for girls (see Hilte et al., 2020c for a detailed interpretation and Prada et al., 2018 for a similar interaction with respect to self-reported usage of and attitudes towards emoticons/emoji).

**FIGURE 2:**
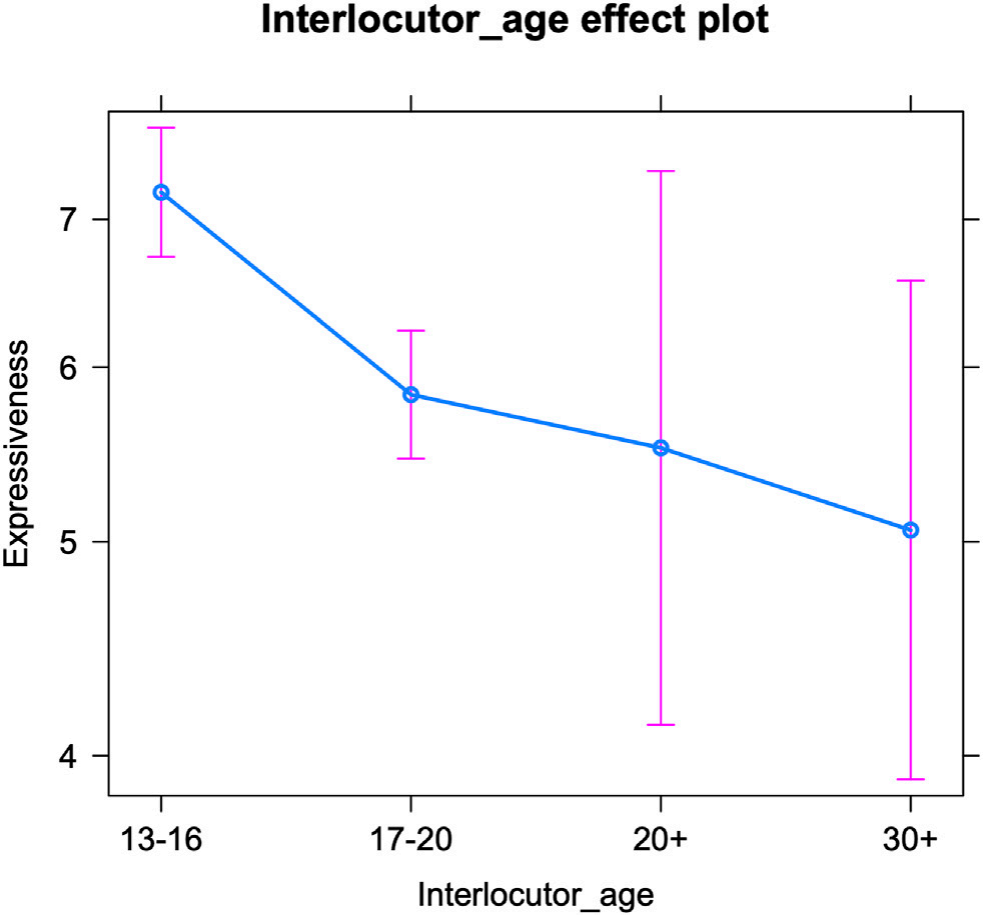
Expressive markers per interlocutor age (predicted counts per 100 tokens).

As Table 3 shows, the effect of generation gap (intra-versus intergenerational communication) is only borderline significant. In order to further inspect this effect, we conducted an additional analysis on the effect of the interlocutors’ age (see Table 4 for the fixed effects and Supplementary Table S2 in the appendix for the Anova). The interlocutors’ age appeared to have a significant impact on the teenage authors’ expressive writing. Figure 2 visualizes a general decrease in expressiveness with increasing interlocutor age: the older the conversation partner, the fewer emoji, allcaps, etc. are inserted by teenagers. This most probably mirrors the interlocutors’ language use, with e.g., emoticon usage decreasing with age (Oleszkiewicz 2017). However, the only significant drops occur between the interlocutor age of 13–16 and 17–20, and between the interlocutor age of 13–16 and 30+. The latter is the least surprising: teenagers appear to (consciously or subconsciously) pick up on older adults’ (e.g., their parents’) less frequent use of these features and mirror this to some extent. But the former decrease indicates that teenagers already adapt their language use to peers who are only a couple of years younger or older than themselves. We know that older teenagers use fewer expressive markers and tend to appreciate them to a lesser extent than younger teenagers (Hilte et al., 2018; Hilte et al., 2019; Hilte et al., 2020c; Verheijen, 2015, 135; Verheijen, 2018, 127). In addition, we have observed a strong awareness among teenagers of this age grading pattern (Hilte et al., 2019). Consequently, this adaptation may not solely consist in unconscious mirroring, but could be based on actual awareness too. Apparently, there is much less awareness with respect to the language use of twenty-somethings, that are neither the teenagers’ peers, nor part of the parent generation. This lower awareness might be related to a limited online contact: the distributions in the corpus (in terms of number of conversations) indicated that teenagers interact the least with twenty-somethings in their instant messaging. Finally, Figure 2 confirms that teenagers’ expressive writing is quite varied when they interact with older interlocutors (20+ and 30+)—we will come back to this in the discussion.

**TABLE 4:** Expressiveness per interlocutor age: Fixed effects^9^.

	Estimate	Standard error	z value	*p* value
(Intercept)	−2.26763	0.05335	−42.508	<2e-16
Interlocutor age (17–20)	−0.21103	0.03567	−5.916	3.31e-09
Interlocutor age (20–30)	−0.26657	0.14861	−1.794	0.072859
Interlocutor age (30+)	−0.35242	0.13303	−2.649	0.008069
Author age (17–20)	−0.33476	0.04765	−7.025	2.13e-12
Author gender (male)	−0.47581	0.05760	−8.261	<2e-16
Author education (technical)	−0.04413	0.05398	−0.817	0.413672
Author education (vocational)	0.16007	0.06194	2.584	0.009764
Number of interlocutors (group chat)	−0.24932	0.06051	−4.120	3.78e-05
Author age (17–20): author gender (male)	0.25048	0.06926	3.617	0.000298

### Orality

**FIGURE 3:**
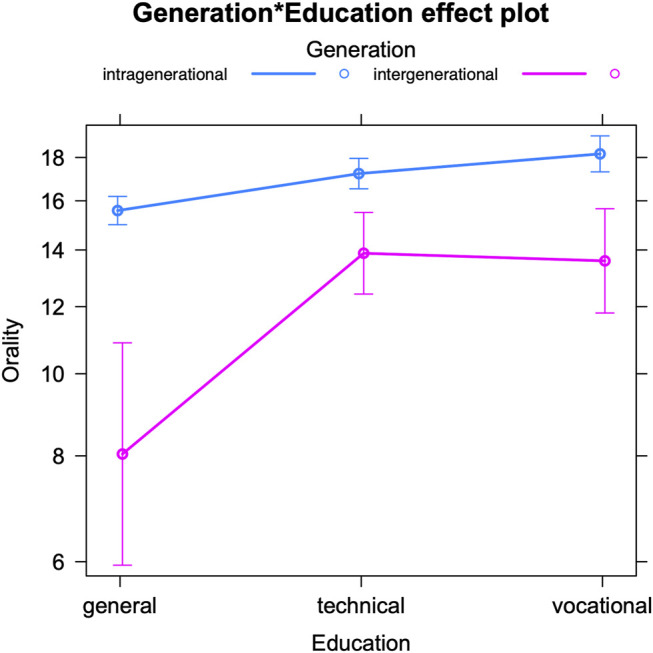
Oral markers in intra-vs. intergenerational talks, by the author’s education (predicted counts per 100 tokens).

The model for orality in intra- and intergenerational talks (see Table 5 for the fixed effects and Supplementary Table S3 in the appendix for the Anova) reveals a significant interaction between the teenage authors’ educational track and the generation gap between interlocutors. The teenagers are predicted to use an average of 16.77 speech-like markers per 100 tokens. But Figure 3 visualizes how all of them, regardless of their educational profile, use significantly fewer oral markers when talking to older interlocutors (intergenerational talks) compared to teenagers (intragenerational talks). This echoes the results for the expressive markers and indicates a mirroring of older interlocutors’ online writing style (Hilte et al., 2020c; Verheijen, 2015, 135; Verheijen, 2018, 127). However, the extent of the drop in oral markers differs per educational track. While this drop is similar for students in more practice-oriented tracks (technical and vocational education), it is much steeper for the most theory-oriented students (general education). This could either point towards a higher sensitivity among these students to the level of formality of conversations, or to a stronger inclination to mirroring. We note that we previously found no evidence for stronger awareness of linguistic age patterns among this group of students (Hilte et al., 2019). So theory-oriented students do not appear to have a higher conscious awareness, but they may still have a stronger subconscious perception of others’ language use as well as a greater tendency to pattern matching. In addition, in view of the different curricula of the three educational tracks, especially with respect to Dutch language teaching (see Hilte et al., 2020a for an overview), a stronger command of standard Dutch might be expected from theory-oriented students. This may facilitate these students’ accommodation with respect to orality since they may have more control of their standard versus speech-like rendition of Dutch words and phrases.

**TABLE 5:** Orality: Fixed effects^10^.

	Estimate	Standard error	z value	*p* value
(Intercept)	−1.84718	0.02445	−75.552	<2e-16
Generation gap (intergenerational)	−0.66168	0.15398	−4.297	1.73e-05
Author education (technical)	0.10040	0.02792	3.596	0.000323
Author education (vocational)	0.15404	0.03113	4.948	7.51e-07
Author age (17–20)	−0.12250	0.02257	−5.427	5.72e-08
Author gender (male)	0.10852	0.02871	3.780	0.000157
Number of interlocutors (group chat)	−0.05558	0.02207	−2.518	0.011789
Generation gap (intergenerational): author education (technical)	0.44532	0.16260	2.739	0.006167
Generation gap (intergenerational): author education (vocational)	0.37092	0.16922	2.192	0.028384
Author age (17–20): author gender (male)	0.11359	0.03311	3.430	0.000603

But how do the findings of the present paper compare to our previous work on gender- and education-based accommodation? In conversations between boys and girls, a weak tendency of mutual convergence could be observed with respect to orality, but this was not statistically significant (Hilte et al., 2020b). In view of the results for age accommodation, we might conclude that teenagers link speech-like markers more to interlocutor age than to gender. In intereducational talks, teenagers significantly altered their degree of speech-like writing depending on their interlocutors’ educational profiles (Hilte et al., forthcoming). However, as opposed to educational adaptation of expressive markers, the adaptation of oral markers could not be interpreted as an accurate mirroring of the interlocutor’s style, which again suggests that oral markers are harder to manipulate (see above). An alternative explanation is that people may not always adapt their language use to their interlocutor’s actual style (i.e., pattern matching), but also to potentially stereotypical (and/or incorrect) images they have about their interlocutor’s style (Hilte et al., forthcoming; Auer et al., 2005, 343). We note that in the present contribution on age-related accommodation, the linguistic adaptation appears accurate and thus 1) either does not support this so-called identity-projection model (Auer et al., 2005, 201, 343) or 2) indicates that the (linguistic) image teenagers have about their conversation partners is, in this case, accurate.

Finally, some patterns relating to the confounding variables emerge from the model. Just like expressive features, oral markers are inserted significantly more often in one-on-one conversations than in group chats, which strengthens our hypothesis that these types of interactions have different conversational dynamics (see also Hilte et al., forthcoming). In addition, a significant interaction between authors’ age and gender emerges. Regardless of their age, boys always write in a significantly more speechlike fashion than girls. But while girls use significantly fewer oral markers as they age, boys do not (for a detailed discussion, see Hilte et al., 2020c).

**FIGURE 4:**
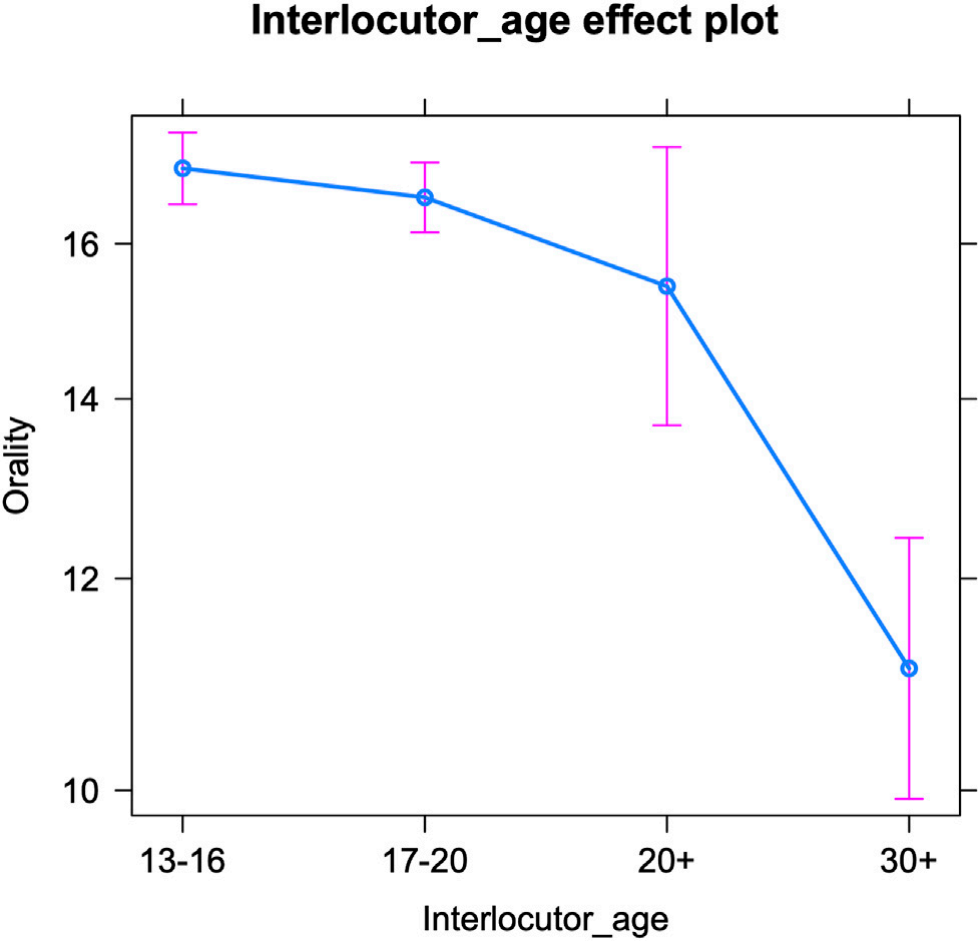
Oral markers per interlocutor age (predicted counts per 100 tokens).

Finally, we examine oral writing per interlocutor age (Table 6 below shows the fixed effects and Supplementary Table S4 in the appendix presents the Anova). The interlocutors’ age significantly influences teenagers’ use of oral markers. Figure 4 shows a similar trend as the one for the expressive markers, i.e., a decrease in orality with increasing interlocutor age: the older the conversation partner, the fewer oral markers (e.g., regional language features) are inserted by teenagers. However, the only age group that is significantly different from (all) others, are people over thirty. There are no significant differences in orality between the other three groups. So teenagers only significantly tone down their speechlike writing when interacting with someone older than thirty. That is quite remarkable, since older teenagers already use significantly fewer oral markers than younger teenagers (Verheijen, 2015, 135; Verheijen, 2018, 127; we note that this pattern was only significant for girls in Hilte et al., 2020c). But while a survey among teenagers revealed a good intuition on age grading among adolescents with respect to expressive markers and (in)correct spelling, the participants did not mention oral vernacular (Hilte et al., 2019). So awareness for younger versus older teenagers’ use of this feature set might be lower. In addition, oral markers may simply be harder to (adequately) adapt than expressive markers, as mentioned above. The significant drop in orality when speaking to people over thirty only, suggests that this age group is not at all associated with speechlike writing in an online context. The change in teenagers’ use of speech-like markers when interacting with this group may be the result of mirroring or may reflect the higher formality of these talks. Finally, just like for expressive markers, a more variable use of oral markers by teenagers can be observed in interactions with older interlocutors (20+ and 30+)—see *Discussion*.

**TABLE 6:** Orality per interlocutor age: Fixed effects^11^.

	Estimate	Standard error	z value	*p* value
(Intercept)	−1.83854	0.02531	−72.639	<2e-16
Interlocutor age (17–20)	−0.02500	0.01631	−1.533	0.125382
Interlocutor age (20–30)	−0.10138	0.06132	−1.653	0.098266
Interlocutor age (30+)	−0.42977	0.05688	−7.556	4.16e-14
Author age (17–20)	−0.12232	0.02287	−5.350	8.82e-08
Author gender (male)	0.10430	0.02882	3.619	0.000295
Author education (technical)	0.10748	0.02801	3.837	0.000125
Author education (vocational)	0.15587	0.03112	5.008	5.49e-07
Number of interlocutors (group chat)	−0.05269	0.02196	−2.400	0.016410
Author age (17–20): author gender (male)	0.11945	0.03305	3.615	0.000301

### Brevity

**FIGURE 5:**
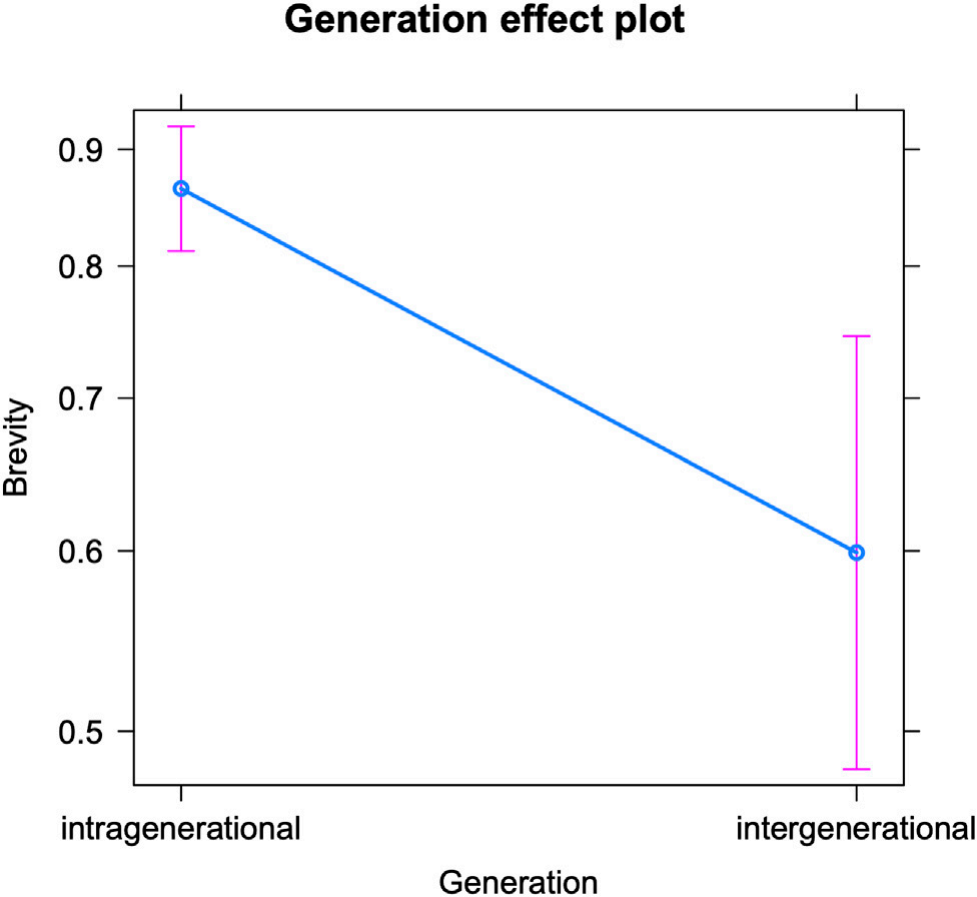
Brevity markers in intra-vs. intergenerational talks (predicted counts per 100 tokens).

We will start by discussing the model for teenagers’ use of brevity markers in intra-versus intergenerational conversations (see Table 7 for the fixed effects and Supplementary Table S5 in the appendix for the Anova). It reveals how the teenagers use an average of 0.85 abbreviations per 100 tokens. But similar to our findings for expressive and oral markers, the teenagers insert significantly more abbreviations and acronyms when interacting with their peers (intragenerational) than with older interlocutors (intergenerational). This is visualized in Figure 5. While these are highly functional markers that show hardly any correlations with the social profile of teenagers in social media writing (e.g., De Decker and Vandekerckhove, 2017, 278), they may actually serve as markers of informality and closeness and therefore be considered less appropriate in online talks with older generations. Moreover, we know that these markers are used most abundantly in (especially early) adolescence, and less frequently as youths grow older (Hilte et al., 2020c; Verheijen, 2015, 135; Verheijen, 2018, 127)—this is also confirmed by the present model. So the observed decrease in brevity seems to be an accurate adaptation to older interlocutors’ online writing style. We note that no comparison can be made between the observed accommodation patterns with respect to brevity markers and our previous findings on gender- and education-based accommodation, since brevity markers were not included as linguistic variables in those two case studies. The recurring finding that teenagers’ use of textisms is more varied in intergenerational talks compared to intragenerational talks (see above), seems to hold for brevity markers too (as the larger confidence interval in Figure 5 shows), and will be discussed below.

**TABLE 7:** Brevity: Fixed effects^12^.

	Estimate	Standard error	z value	*p* value
(Intercept)	−4.76681	0.06404	−74.430	<2e-16
Generation gap (intergenerational)	−0.36773	0.10968	−3.353	0.000800
Author age (17–20)	−0.17227	0.04056	−4.247	2.17e-05
Author gender (male)	0.31996	0.09046	3.537	0.000404
Author education (technical)	−0.01214	0.09157	−0.133	0.894506
Author education (vocational)	0.29453	0.09690	3.040	0.002370
Author gender (male): author education (technical)	−0.30486	0.13385	−2.278	0.022753
Author gender (male): author education (vocational)	−0.40278	0.14841	−2.714	0.006647

The model also reveals patterns relating to the confounding factors of author gender and education, with most abbreviations being produced by girls in vocational education and by boys in vocational and general education (see also Hilte et al., 2020c).

**FIGURE 6:**
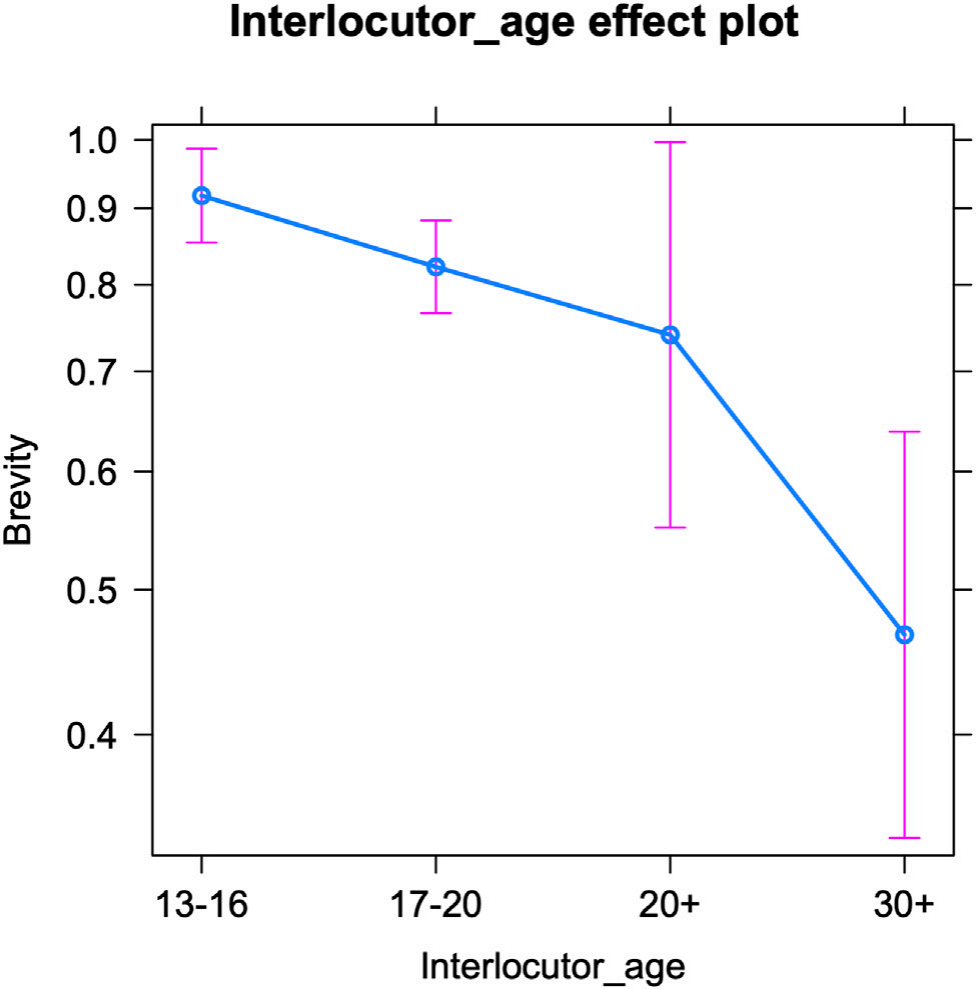
Brevity markers per interlocutor age (predicted counts per 100 tokens).

Finally, we examine the use of brevity markers per interlocutor age (Table 8 below shows the fixed effects and Supplementary Table S6 in the appendix presents the Anova). The interlocutors’ age significantly influences teenagers’ use of abbreviations and acronyms. Figure 6 visualizes a pattern similar to that of expressive and oral markers, i.e., a decrease in brevity features with increasing interlocutor age: the older the conversation partner, the fewer brevity markers are used by teenagers. All (interlocutor) age groups significantly differ from each other in this respect, except for twenty-somethings (20–30), who overlap much with the other categories. But in general, teenagers seem aware of the (supposedly) less frequent use of abbreviations by their older interlocutors and appear to mirror this. A final conclusion to draw from this model and figure, is that the teenagers’ use of brevity markers shows much more variance in interactions with older interlocutors (20+ and 30+)—see below for an extensive discussion.

**TABLE 8:** Brevity per interlocutor age: Fixed effects^13^.

	Estimate	Standard error	z value	*p* value
(Intercept)	−4.71184	0.06544	−72.001	<2e-16
Interlocutor age (17–20)	−0.10977	0.03662	−2.997	0.002725
Interlocutor age (20–30)	−0.21457	0.15182	−1.413	0.157572
Interlocutor age (30+)	−0.67674	0.15926	−4.249	2.14e-05
Author age (17–20)	−0.15911	0.04045	−3.933	8.38e-05
Author gender (male)	0.30470	0.08953	3.403	0.000666
Author education (technical)	−0.00185	0.09077	−0.020	0.983743
Author education (vocational)	0.28581	0.09598	2.978	0.002902
Author gender (male): author education (technical)	−0.30371	0.13252	−2.292	0.021918
Author gender (male): author education (vocational)	−0.39073	0.14678	−2.662	0.007768

## Discussion

This study contributes to research on accommodation in intergenerational communication by focusing on an under-researched target group (teenagers) and interactional setting (spontaneous written online conversations). In addition, it extends our own previous work on online accommodation from the social variables of interlocutors’ gender and education to their age.

The distributions in the corpus indicate that teenagers’ instant messaging primarily proceeds within peer group networks and much less frequently across generations. But despite this more peripheral online contact with older interlocutors, the teenagers do adapt their writing style to them. We examined linguistic features relating to the chatspeak maxims of expressive compensation (e.g., emoji), orality (e.g., regional language features), and brevity (e.g., abbreviations). The same trend emerges for all three feature sets: teenagers insert significantly fewer expressive, oral, and brevity markers in intergenerational talks (including older people) than in intragenerational talks (peers only). This change may be related to differences in the relationship between interlocutors: all of the features may be considered markers of informality, closeness and peer-group talk and therefore deemed less appropriate when chatting with interlocutors of older generations, even if these are relatives.

At the same time the decrease in the different types of chatspeak features can also be the result of straightforward mirroring: (especially young) teenagers are more ardent users of these features than older interlocutors (Hilte et al., 2018; Hilte et al., 2020c; Oleszkiewicz et al., 2017; Prada et al., 2018; Verheijen 2015; 2018), so in intergenerational contexts, they seem to accurately adapt their writing style to older interlocutors by inserting these markers less frequently. We recall that mirroring or convergence narrows the linguistic and therefore (according to CAT) also the social distance between conversation partners and is generally evaluated positively (Burgoon et al., 2017; Dragojevic et al., 2015, 13, 15). Our present research shows that the desire for social approval (a driving force behind accommodation) does not only hold among peers, but also across generations. In addition, youths accommodating (or even overaccommodating) to older interlocutors is in line with previous findings on (spoken) intergenerational communication (Williams and Nussbaum 2001, 85, 89; Giles and Gasiorek 2011, 233–234). Consequently, these findings for spoken dialogue seem to hold in written online conversations too, although overaccommodation does not seem to be an issue here, since it seems likely that dropping the use of typical markers of the online genre in order to accommodate to the online writing style of older interlocutors would not be perceived as irritating or patronizing. Furthermore, the nature of the linguistic variables subject to mirroring is informative too. Recall how CAT allows for both conscious and unconscious mirroring (Dragojevic et al., 2015). The teenagers’ adaptation of these more intentional digital language features or textisms offers an example of more deliberate accommodation (see also Adams et al., 2018).

While the accommodation pattern for expressive compensation and the use of brevity markers is not impacted by the teenagers’ sociodemographic profiles, the observed convergence with respect to the use of orality markers differs depending on the teenagers’ educational track, with stronger adaptation by theory-oriented students. Since these students have not proven to be more (consciously) aware of age-related linguistic variation (Hilte et al., 2019), they might have a stronger inclination to subconsciously match their language use to their conversation partner’s. Such matching or mirroring can be considered the product of meta-linguistic skills that are actually part of language teaching in more theory-oriented tracks only (VVKSO, 2014, 5–6). In addition, a stronger command of standard Dutch–which can be expected from theory-oriented students (see above)—might also increase these students’ control of their standard versus speech-like rendition of Dutch words and phrases.

Besides the teenagers’ sociodemographic profiles, we inspected a final confounding factor: the number of interlocutors in the conversation. The teenagers’ mirroring of older interlocutors’ writing style is not different in one-on-one chats compared to group chats. That is quite striking, since we might expect stronger convergence in one-on-one talks: they are often of a more intimate nature than group chats and trust is said to facilitate communicative convergence (Riordan et al., 2013). In addition, mirroring is more straightforward when there is only one interlocutor to mirror. In our previous work, gender convergence appeared more outspoken in one-on-one conversations (for expressive markers; Hilte et al., 2020b), but education convergence did not (Hilte et al., forthcoming). While it is hard to pinpoint the exact determinants, gender accommodation might be more personal and intimate than adaptation based on interlocutors’ age or educational track. Furthermore, it should be noted that mirroring in intergenerational talks (and in intereducational talks, respectively) is in a sense less obvious than in mixed-gender contexts, because interlocutors from different generations (or with distinct educational profiles, respectively) often have quite different social/cultural backgrounds too and tend to have less frequent online contact compared to teenage boys versus girls (see also Hilte et al., forthcoming). Still, both the educational background and the age of the interlocutor trigger accommodation.

Apart from the analyses on intra-versus intergenerational talks, we examined teenagers’ online writing style by interlocutor age, i.e., by treating age as a categorical variable with four levels: 13–16, 17–20, 20–30, and 30+. The teenagers’ use of orality, brevity and expressive markers decreases as the interlocutors’ age increases. So the older the conversation partner is, the fewer of these features teenagers use. However, some subtle differences can be noted between the three sets of linguistic variables. For brevity markers, a gradual decrease in frequency emerges with increasing interlocutor age. For expressive markers, the decrease is only significant moving from the interlocutor age of 13–16 (early adolescence) to 17–20 (later adolescence) and to people over thirty, whereas for oral markers, teenagers’ style only significantly changes in interactions with people over thirty. Both the frequency of expressive markers and that of oral markers drop from the age of seventeen onwards (Hilte et al., 2018; Hilte et al., 2020c), but apparently, only the former is deemed crucial to adapt when teenagers interact with peers in later adolescence. Compared to oral markers, expressive markers might be regarded as more childish by older adolescents and thus have a more negative connotation. An alternative explanation is that expressive markers are simply more “visible” features, that are inserted deliberately, making them easier to manipulate and thus more prone to accommodative change. Oral features may not only be harder to adapt but also to detect (see above). The decreasing frequency of expressive markers in adolescent social media writing guarantees a better match for older adolescents’ writing style and shows that accommodative changes also follow the ‘adolescent peak’ that was attested repeatedly in teenagers’ (on- and offline) language use: i.e., an increase in the use of non-standard features (whether oral vernacular or digital vernacular), peaking mid-puberty and then decreasing again (see above). Finally, the drop in the use of oral markers in conversations with people over thirty may be most telling too. Teenagers might indeed be well aware of the fact that speech-like writing is very common up to a certain age. People in their twenties may still be used to it, but older generations may be perceived as producing less speech-like writing and as adhering more closely to standard grammar.

Finally, recall how more variance was consistently observed in the teenagers’ use of expressive, oral and brevity markers when conversing with older interlocutors (20+) compared to when conversing with peers (13–20). Several potential explanations come to mind. A first hypothesis concerns sample size: the dataset contains fewer intergenerational conversations, as the platforms of Facebook Messenger and WhatsApp proved to be highly peer-oriented. The smaller size of the intergenerational subset may lead to more uncertainty in the statistical models and thus result in a larger confidence interval. In addition, more variation can be expected in intergenerational settings, since more interlocutor ages are grouped by this label (all non-teenage interlocutors, i.e., everyone older than 20) than by the intragenerational label (only teenage interlocutors, i.e., aged 13–20). A final potential explanation concerns accommodation. Without the corresponding data from the older (i.e., non-adolescent) interlocutors, we cannot investigate whether or not this wider variance in the teenagers’ writing style reflects accommodation to a given (specific) interlocutor’s online writing style. However, these findings do suggest that there is not one singular way in which teenagers adapt their online communication to that of people belonging to these older age groups, but that they are rather doing some level of adaptation for the particular person or context. In future work, this can be investigated (and more statistical power can be gained) by adding more data for teenagers interacting with older interlocutors.

Another limitation of the present contribution is that the (a)symmetry of convergence by younger and older interlocutors in intergenerational communication could not be examined. Because the corpus only contains teenagers’ instant messages, we cannot analyze the older interlocutors’ writing style nor their (potential) adaptations to adolescent writing. So in this respect, we cannot complement our previous results on education accommodation (symmetric convergence) and gender accommodation (asymmetric convergence, stronger for boys). This should be examined in future work.

In addition, the temporal dimension of accommodation falls outside the scope of this paper. Our findings can be complemented with a study on the diachrony of the observed accommodative adjustments in order to see when patterns of convergence emerge in an interaction and how they evolve.

Finally, these findings (along with our previous findings on gender and education accommodation) on the “production” of accommodation can be complemented with a study on the “perception” of the phenomenon. Are teenagers aware of linguistic mirroring? And how do they perceive/appreciate conversations in which mirroring does (not) occur?

Still, the present study convincingly demonstrates that age differences trigger accommodation in adolescent social media writing. Furthermore, we see different patterns for different types of features: while expressive features are prototypical markers of the genre, the abundant or (perceived) excessive use of these features apparently is considered inappropriate as soon as the interlocutor is beyond early adolescence. Oral markers, however, need not be suppressed when communicating with young adults, but they may be felt less appropriate for communication with “older” adults. And finally, the use of brevity markers (that may need some decoding) is gradually reduced by teenagers according to the increasing age of their interlocutors. These patterns point to a general awareness of age-appropriate writing among these teenagers, not only in terms of their own socio-demographic profile but also in terms of that of their interlocutors.

## Data Availability

In order to protect the participants’ privacy, and following the guidelines of our university’s ethical committee, the collected dataset cannot be made publicly available. For more information on the database, see chapter 1 in https://repository.uantwerpen.be/docman/irua/948a9a/159941.pdf.

## Footnotes

1 This phenomenon is also referred to as e.g., alignment, matching, or synchrony, depending on the scientific field (see Burgoon et al., 2017 for an overview).

2 A token is a visual unit in a text, separated by whitespaces. In this corpus of instant messages, a token can be a word, but also e.g., an emoji or isolated punctuation mark.

3 We deleted conversations that included interlocutors with an unknown age, or interlocutors from more than two different age groups (e.g., group chats between teenagers, twenty-somethings and people over thirty).

4 The number of participants in the different subgroups adds up to a higher number than the total number of participants, as the same author may occur in the corpus in both age categories (i.e., if they donated older parts of their chat history too). In addition, participants can occur in multiple conversational settings. For instance, the same teenager may participate in interactions among teenagers and in interactions with older people. Finally, the number of conversations including younger versus older teenagers adds up to a higher number than the total number of conversations since some interactions include both younger and older teenagers.

5 Note that gifs and images (including e.g., memes) could not be included as expressive markers, since they were automatically deleted from the chat submissions during the data collection procedure (see *Materials*).

6 With the exception of e.g., certain oral markers: one may wonder to what extent people producing speech-like features in their online writing are actually aware of the standard equivalent and thus deliberately opt for a “non-standard”, oral, variant.

7 As implemented in the R-package “lme4” (Bates et al., 2017).

8 Reference category: 13- to 16-year-old girls in general education, interacting one-on-one with teenagers (intragenerational).

9 Reference category: 13- to 16-year-old girls in general education, interacting one-on-one with teenagers aged 13–16.

10 Reference group: 13- to 16-year-old girls in general secondary education, interacting one-on-one with other teenagers (intragenerational).

11 Reference category: 13- to 16-year-old girls in general education, interacting one-on-one with other teenagers aged 13–16.

12 Reference category: 13- to 16-year-old girls in general education, interacting one-on-one with other teenagers (intragenerational).

13 Reference category: 13- to 16-year-old girls in general education, interacting one-on-one with other teenagers aged 13–16.
