# Supplementary material for: Interlocutors’ Age Impacts Teenagers’ Online Writing Style: Accommodation in Intra- and Intergenerational Online Conversations
Source: Front Artif Intell. 2021 Aug 30;4:738278. doi: 10.3389/frai.2021.738278 (PMC8435778; doi:10.3389/frai.2021.738278)
Supplement: Supplementary file 1 [file DataSheet1.docx]

**Appendix**

**Supplementary tables**

|  | **Chi-squared value** | **Degrees of freedom** | **p value** |
| --- | --- | --- | --- |
| Generation gap | 3.9363 | 1 | 0.0472549 |
| Author age | 32.0318 | 1 | 1.517e-08 |
| Author gender | 49.7051 | 1 | 1.787e-12 |
| Author education | 14.5628 | 2 | 0.0006882 |
| Number of interlocutors | 20.0861 | 1 | 7.403e-06 |
| Author age : author gender | 13.1665 | 1 | 0.0002850 |

Table S1. Expressiveness: Anova

|  | **Chi-squared value** | **Degrees of freedom** | **p value** |
| --- | --- | --- | --- |
| Interlocutor age | 39.599 | 3 | 1.296e-08 |
| Author age | 36.698 | 1 | 1.379e-09 |
| Author gender | 57.024 | 1 | 4.306e-14 |
| Author education | 11.535 | 2 | 0.0031275 |
| Number of interlocutors | 16.978 | 1 | 3.781e-05 |
| Author age : author gender | 13.081 | 1 | 0.0002983 |

Table S2. Expressiveness per interlocutor age: Anova

|  | **Chi-squared value** | **Degrees of freedom** | **p value** |
| --- | --- | --- | --- |
| Generation gap | 43.2241 | 1 | 4.882e-11 |
| Author education | 28.5730 | 2 | 6.244e-07 |
| Author age | 17.6878 | 1 | 2.603e-05 |
| Author gender | 45.3530 | 1 | 1.645e-11 |
| Number of interlocutors | 6.3423 | 1 | 0.011789 |
| Generation gap : author education | 7.5940 | 2 | 0.022437 |
| Author age : author gender | 11.7666 | 1 | 0.000603 |

Table S3. Orality: Anova

|  | **Chi-squared value** | **Degrees of freedom** | **p value** |
| --- | --- | --- | --- |
| Interlocutor age | 58.7798 | 3 | 1.071e-12 |
| Author age | 15.6796 | 1 | 7.503e-05 |
| Author gender | 43.8356 | 1 | 3.571e-11 |
| Author education | 28.6584 | 2 | 5.983e-07 |
| Number of interlocutors | 5.7584 | 1 | 0.0164097 |
| Author age : author gender | 13.0648 | 1 | 0.0003009 |

Table S4. Orality per interlocutor age: Anova

|  | **Chi-squared value** | **Degrees of freedom** | **p value** |
| --- | --- | --- | --- |
| Generation gap | 11.2409 | 1 | 0.0008002 |
| Author age | 18.0383 | 1 | 2.165e-05 |
| Author gender | 4.0468 | 1 | 0.0442554 |
| Author education | 13.1858 | 2 | 0.0013701 |
| Author gender : author education | 9.0046 | 2 | 0.0110836 |

Table S5. Brevity: Anova

|  | **Chi-squared value** | **Degrees of freedom** | **p value** |
| --- | --- | --- | --- |
| Interlocutor age | 25.4042 | 3 | 1.271e-05 |
| Author age | 15.4700 | 1 | 8.383e-05 |
| Author gender | 3.3257 | 1 | 0.06820 |
| Author education | 11.9045 | 2 | 0.00260 |
| Author gender : author education | 8.8354 | 2 | 0.01206 |

Table S6. Brevity per interlocutor age: Anova
